# The effects of music listening on pain and stress in the daily life of patients with fibromyalgia syndrome

**DOI:** 10.3389/fnhum.2015.00434

**Published:** 2015-07-30

**Authors:** Alexandra Linnemann, Mattes B. Kappert, Susanne Fischer, Johanna M. Doerr, Jana Strahler, Urs M. Nater

**Affiliations:** ^1^Department of Psychology, University of MarburgMarburg, Germany; ^2^Institute of Psychiatry, Psychology and Neuroscience, King’s College LondonLondon, UK

**Keywords:** ecological momentary assessment, fibromyalgia syndrome, music listening, pain, stress

## Abstract

Music listening is associated with both pain- and stress-reducing effects. However, the effects of music listening in daily life remain understudied, and the psycho-biological mechanisms underlying the health-beneficial effect of music listening remain unknown. We examined the effects of music listening on pain and stress in daily life in a sample of women with fibromyalgia syndrome (FMS; i.e., a condition characterized by chronic pain) and investigated whether a potentially pain-reducing effect of music listening was mediated by biological stress-responsive systems. Thirty women (mean age: 50.7 ± 9.9 years) with FMS were examined using an ecological momentary assessment design. Participants rated their current pain intensity, perceived control over pain, perceived stress level, and music listening behavior five times per day for 14 consecutive days. At each assessment, participants provided a saliva sample for the later analysis of cortisol and alpha-amylase as biomarkers of stress-responsive systems. Hierarchical linear modeling revealed that music listening increased perceived control over pain, especially when the music was positive in valence and when it was listened to for the reason of ‘activation’ or ‘relaxation’. In contrast, no effects on perceived pain intensity were observed. The effects of music listening on perceived control over pain were not mediated by biomarkers of stress-responsive systems. Music listening in daily life improved perceived control over pain in female FMS patients. Clinicians using music therapy should become aware of the potential adjuvant role of music listening in daily life, which has the potential to improve symptom control in chronic pain patients. In order to study the role of underlying biological mechanisms, it might be necessary to use more intensive engagement with music (i.e., collective singing or music-making) rather than mere music listening.

## Introduction

Music listening has been shown to alleviate pain. Thus, it would appear to be a promising means of symptom reduction and health promotion, given that it is an activity of daily life that is popular, cost-effective and easily accessible. However, the mechanisms underlying its potential pain-reducing effect remain to be elucidated. We posit that the pain-reducing effect of music is mediated by a reduction in the activity of stress-responsive systems in the body.

Music listening is associated with reduced subjective stress levels and affects physiological markers of stress (Pelletier, 2004; Chanda and Levitin, 2013; Koelsch, 2014). Koelsch (2014) proposes a model of music-evoked emotions in which music is initially processed in the central nervous system, then further impacts endocrine, autonomic and immune activity, and leads to the experience of a broad range of emotions. A stress-reducing effect of music listening may be explained by music particularly affecting activity in the hippocampal formation, which in turn influences the activity of the hypothalamus–pituitary–adrenal (HPA) axis (Koelsch, 2014). The HPA axis constitutes one of the major stress-responsive systems in the body, and its activation leads to the secretion of the steroid hormone cortisol (Hellhammer et al., 2009). Research has linked music listening to a down-regulation of HPA axis activity, as mirrored in lower concentrations of cortisol (Chanda and Levitin, 2013). This has led to the conclusion that the health-beneficial effect of music listening is mediated by a reduction in stress (Thoma and Nater, 2011). Another stress-responsive system of high relevance is the autonomic nervous system (ANS). Music listening has also been associated with a down-regulation of ANS activity, which is reflected by both lower blood pressure and lower heart rate (Kreutz et al., 2012).

Interestingly, stress has been discussed as an etiological factor in the manifestation of chronic pain (Fitzcharles and Yunus, 2012), giving rise to the conclusion that music listening has the capacity to positively influence pain. Indeed, the term ‘audio-analgesia’ was coined in this context (Gardner and Licklider, 1959). Nevertheless, the exact mechanisms underlying the pain-reducing effect of music remain unclear. One open question concerns whether music listening can reduce pain *per se* (i.e., direct effect) or whether it facilitates coping with pain (i.e., indirect effect). Bernatzky et al. (2012) state that music exerts effects in the brain that directly impact on the relevant pain circuits, which in turn reduce the perception of pain intensity. However, the empirical evidence is not consistent in this regard, as there are also studies showing no music-induced reduction in perceived pain intensity (i.e., MacDonald et al., 2003). Similarly, Mitchell and MacDonald (2006) did not find a reduction in pain intensity, but did find an increase in control over pain, and thus propose that music listening is successful in reducing pain by specifically improving control over pain (Mitchell and MacDonald, 2012). While the exact mechanisms remain unclear, it has been discussed that improved control over pain may be achieved via distraction from pain or via induced relaxation (MacDonald et al., 2003; Mitchell and MacDonald, 2006; Mitchell et al., 2006).

When experiencing pain, information is forwarded from nociceptive receptors in the periphery of the body to cortical areas in the brain. Different brain mechanisms process information either serially or in parallel, with certain brain circuits being involved in the sensory-discriminative components of pain and others being involved in the affective-motivational components of pain (Treede et al., 1999). Research examining the neurobiological mechanisms underlying the pain-reducing effect of music identified the limbic system – which is involved in the affective-motivational modulation of pain – as a key structure in the brain that is affected by listening to music (Bernatzky et al., 2012). As the cortico-limbic pathways exert an inhibitory effect on pain, it is likely that music listening exerts a pain-reducing effect. At the same time, the limbic system, especially the hippocampal formation, is closely associated with the modulation of the HPA axis (Jankord and Herman, 2008). In this way, music listening is thought to exert a stress-reducing effect (Koelsch, 2014). Bernatzky et al. (2012, p. 270) state in this regard that a ‘hypothalamic changeover’ leads to music-induced distraction and relaxation in the context of pain. In other words, on a neurobiological level, music listening exerts effects in the central nervous system that are critical to the modulation of both pain and stress. The limbic system can be regarded as a key structure in this context, which further impacts on neuroendocrine and autonomic functioning.

However, most of the studies on music listening and pain are based on studies of acute pain in the clinical context. What remains understudied so far is the effect of music listening in chronic pain conditions (Finlay, 2014). In contrast to acute pain, which requires a pain stimulus to be present, the perception of pain in chronic pain occurs even though no current pain stimulus is present (Lee et al., 2011). This persistent experience of pain is explained by long-lasting alterations in both the central and the peripheral nervous system as nociceptive information transmission is impaired (Lee et al., 2011). Therefore, patients with chronic pain show an altered processing of sensory input, which could be linked to an effect on the limbic system (Bennett, 1999). Inhibitory effects of the thalamus on limbic structures seem to be impaired, thereby increasing the experience of pain (Bennett, 1999; Tracey and Mantyh, 2007). As chronic pain – in contrast to acute pain – is associated with these physiological alterations, it is of utmost importance to examine whether music listening can affect pain in chronic pain conditions as well.

Fibromyalgia syndrome (FMS) is a medically unexplained condition mainly characterized by chronic widespread pain impacting heavily on patients’ quality of life (Woltman et al., 2012). Stress seems to play a major role in the manifestation of pain symptoms in FMS patients: it was demonstrated that patients with FMS show alterations in HPA axis (Tak et al., 2011) as well as in ANS functioning (Martinez-Lavin, 2002). We previously investigated the role of both HPA axis and ANS in the relationship between stress and pain in daily life (Fischer et al., in revision). Hereby, we have shown that stress exacerbates pain in FMS patients, with HPA axis activity (but not ANS activity) being associated with pain. As music listening has been shown to reduce stress and stress system activity, the question arises whether music listening can also have a positive impact on pain in FMS patients. The still limited number of studies point toward a pain-reducing effect of music listening in these patients (Onieva-Zafra et al., 2013; Garza-Villarreal et al., 2014). In their experimental study, Garza-Villarreal et al. (2014) found pain-reducing effects, which they hypothesized to be due to cognitive and emotional processes, as music listening might have helped to distract from the pain and lead to a state of relaxation. However, the study was set in a highly artificial laboratory context, thus limiting the generalizability of its results. Onieva-Zafra et al. (2013) conducted an intervention study set in daily life, in which FMS patients were instructed to listen to pre-recorded music CDs almost daily for 14 consecutive days. Although music listening was associated with alleviated pain intensity levels, the underlying mechanisms remain unclear, as no biological markers were assessed in this study.

Taken together, the currently available literature suggests that music listening has pain- and stress-reducing effects. The underlying mechanisms, however, remain to be elucidated. Since most of the previous studies were set in experimental contexts examining acute pain, an investigation of the psycho-biological effects of music listening on chronic pain in daily life is warranted.

### Research Question

Our overarching research question is whether patients with FMS benefit from mere music listening in daily life, and what the mechanisms underpinning the potential health-beneficial effect of music listening are. Based on the evidence summarized above, we hypothesized that music listening reduces pain intensity and increases control over pain (Hypotheses 1 and 2), that music listening has a stress-reducing effect both on subjective stress levels and markers of HPA and ANS activity (Hypothesis 3), and that these biomarkers mediate the pain-reducing effect (Hypothesis 4).

## Materials and Methods

### Participants

A total of 30 women meeting the Fibromyalgia Research Criteria (Wolfe et al., 2011; mean age = 50.7 ± 9.9 years, range: 27–64 years) were recruited via specialized clinics and various advertising outlets from the general population (i.e., newspapers, self-help groups, internet) as part of a bigger study on everyday life stress in patients with functional somatic symptoms. The eligibility criteria were as follows: diagnosis of fibromyalgia according to the Fibromyalgia Research Criteria, female sex (due to the majority of FMS patients being female, Wolfe et al., 1995), German as native language, age between 18 and 65 years, body mass index (BMI) in the range of 18–30, regular menstrual cycle or being post-menopausal, no acute or chronic unmedicated conditions influencing biological stress markers, no current pregnancy or breast-feeding, no lifetime psychotic or bipolar disorder, no eating disorder within the past five years, no substance abuse within the past two years, and no current episode of major depression. As compensation, participants received 80 Euro. The participants were informed about the aims of the study and gave written informed consent. The study was approved by the Ethics Committee of the Department of Psychology at the University of Marburg, Germany.

### Procedure

The study was designed as an ecological momentary assessment study (Shiffman et al., 2008). Participants were examined on 14 consecutive days. Initially, potential participants underwent a telephone-based interview in order to check the eligibility criteria. If they fulfilled the criteria, they received a detailed study description by post. Subsequently, participants were invited to the laboratory for an introductory session. For the duration of the study, patients received an iPod^®^ touch (Apple, Cupertino, CA, USA), on which they were required to complete six daily assessments on each day by means of the software iDialogPad (G. Mutz, University of Cologne, Germany). The participants were instructed to switch on the iPod^®^ upon waking up the following morning, not to turn it off from then on, and to recharge it every night. The assessment period began on the day following the introductory session. Each day, participants were asked to trigger the first assessment themselves directly after awakening. An activated timer prompted participants to complete a second set of questions 30 min afterwards (to determine the cortisol awakening response, which was not used in the current analyses). Further fixed assessments followed at 11.00, 14.00, 18.00, and 21.00 h. Participants were instructed to provide a saliva sample after each assessment for the later analysis of salivary cortisol and salivary alpha-amylase, for which they were provided with pre-labeled tubes. After completing the 14 days of assessment, the participants returned to the laboratory to hand over the saliva samples and iPod^®^. On this occasion, they also completed online-based questionnaires. Additionally, a post-monitoring interview was conducted, checking for problems in handling the iPod^®^ or problems with the collection of saliva samples and asking for any unusual events during the measurement period.

### Measures

#### Ecological Momentary Assessment Items

At all assessments, except for directly after awakening, participants had to complete items regarding their music listening behavior, their momentary pain experience, and their momentary stress level.

##### Music listening behavior

Participants were asked whether they had deliberately listened to music since the last assessment, which was defined as actively deciding to listen to music (i.e., by turning on the radio or a music listening device). If they reported deliberate music listening, they were then required to answer further items concerning their listening to music. They were asked to rate the perceived valence on a visual analog scale (VAS) ranging from 0 (‘sad’) to 100 (‘happy’) and the perceived arousal on a VAS ranging from 0 (‘relaxing’) to 100 (‘energizing’). In the context of music, pain, and stress, the dimensions of valence and arousal are of special relevance. While the kind of music that is effective in reducing pain is still subject to debate, there is accumulating evidence that music which is positive in valence is especially effective, independent of its arousal (Roy et al., 2008). However, in the context of stress, the reverse pattern seems to be true, with music which is low in arousal being especially effective in reducing stress (Kreutz et al., 2012; Chanda and Levitin, 2013). Based on the occurrence of deliberate music listening, time points were classified as ‘music episodes’ if deliberate music listening was reported.

Subsequently, participants were asked to indicate the reasons why they had listened to music by choosing from among the following options: ‘relaxation,’ ‘activation,’ ‘distraction,’ and ‘reducing boredom.’ These have been stated as the most common reasons for listening to music in previous studies (Juslin et al., 2008; Linnemann et al., 2015b). Further, in the context of pain, it has been long discussed whether music listening is effective in reducing pain by distracting from pain and/or by inducing relaxation (Mitchell and MacDonald, 2012). This therefore highlights the importance of investigating the link between reasons for music listening and potential pain- and stress-reducing effects.

##### Pain

Perceived pain intensity (Myles et al., 1999) and control over pain (Haythornthwaite et al., 1998) were measured as indicators for the experience of pain. Perceived pain intensity was measured using a VAS ranging from 0 (‘At the moment, I am in no pain’) to 100 (‘At the moment, I am in the most intense pain possible’). Perceived control over pain was measured using the item ‘I had the feeling that I was in control of the pain,’ which was rated on a 6-point Likert scale ranging from 0 to 5, with low values indicating low control over pain and high values indicating high control.

##### Stress

###### Subjective stress

Subjective stress was measured using the item ‘At the moment, I feel stressed,’ which was rated on a 5-point Likert scale ranging from 0 (‘not at all) to 4 (‘very much’). In order to keep the number of items asked to a minimum, we decided to use a single- item measure for stress, which proved to have sufficient psychometric qualities (Elo et al., 2003).

###### Physiological stress

After each assessment, participants were asked to collect a saliva sample for the later analysis of salivary cortisol (sCort) and salivary alpha-amylase (sAA) which we measured as biomarkers of stress. High cortisol levels generally indicate high levels of stress (Hellhammer et al., 2009). The secretion of cortisol follows a marked diurnal rhythm, with a rise in the morning and a subsequent decrease of cortisol towards the evening. sAA is an enzyme which is secreted from the salivary glands in the oral cavity. As its secretion is regulated by the ANS, sAA is also regarded as an indirect indicator of autonomic activation (Nater and Rohleder, 2009). Like cortisol, alpha-amylase follows a distinct diurnal pattern, with a decrease within 60 min after awakening and a steady increase of activity during the course of the day. Both sCort and sAA can be considered as valid physiological markers of stress system activity (Nater et al., 2013).

Measures of sCort and sAA were obtained from the unstimulated whole saliva samples collected during the 14-day measurement period. The participants were instructed to collect the samples as follows: first, they should rinse their mouth with water and then swallow all remaining saliva before collecting saliva for 2 min, which they should then transfer into SaliCap^®^ tubes via a straw. They were also asked to refrain from eating, smoking, brushing their teeth, or drinking anything but water for 1 h prior to sample collection. Participants were asked to store samples in a freezer or a fridge as soon as possible at home. Upon returning to the laboratory, the samples were stored at -20°C at the Biochemical Laboratory of the Department of Psychology, University of Marburg, until analysis. sCort levels were measured using a commercially available enzyme-linked immunoassay (IBL, Hamburg, Germany). sAA activity was measured using a kinetic colorimetric test and reagents obtained from Roche (Fa. Roche Diagnostics, Mannheim, Germany). Inter- and intra-assay variance for both sCort and sAA was below 10%.

#### Paper–Pencil Questionnaires

During the introductory session, participants were asked to complete a questionnaire on their socio-demographic background. Furthermore, after having completed the study protocol, participants completed the music preference questionnaire once online (MPQ; Nater et al., 2005). The MPQ provides insight into participants’ habitual music listening behavior and is based on their subjective experience of their music listening behavior. The first item covers the preference for the most common music genres (i.e., ‘pop,’ ‘rock,’ ‘classical music’) by asking participants to indicate which music genre they prefer, using a scale ranging from 1 (‘not at all’) to 5 (‘very much’). Subsequent items evaluate common reasons for music listening (i.e., ‘relaxation,’ ‘activation,’ ‘distraction), with participants being asked to indicate how frequently they listen to music for the respective reasons on a scale from 1 (‘never’) to 5 (‘very often’). Further items cover participants’ music-making experience (i.e., information about playing an instrument or singing in a choir). Finally, the personal significance of music for participants is stated on a scale ranging from 1 (‘not important’) to 5 (‘very important’).

### Statistical Analyses

In order to account for the nested structure of the data, analyses were performed using two-level hierarchical linear modeling (HLM; Raudenbush et al., 2004). Perceived pain intensity, perceived control over pain, subjective stress, sCort, sAA, and items on music listening such as music listening (yes/no), valence, arousal, and reasons for music listening were considered as level-1 variables. Furthermore, at the momentary level (level-1), the time of day was entered into analyses concerning the relationship between biological parameters and music listening due to the known diurnal patterns of both sCort and sAA. However, as only time points at which music listening was reported were entered into analyses concerning valence/arousal and reasons for music listening (thus reducing the number of level-1 observations), we did not include time of day as predictor in these analyses. Exploratory analyses revealed that entering time of day as predictor did not alter the results. At the individual level (level-2), the intercept (β_0_) was modeled as a function of number of music episodes (γ_01_) and a residual component (u_0_). In analyses concerning biological parameters, both age and BMI were entered additionally at the individual level (level-2) due to their known associations with HPA axis and ANS regulation.

The total number of music episodes varied between 0 and 53 per participant per measurement period, with participants listening to music once per day on average (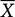 = 13.80 ± 13.34). Therefore, we controlled for the total number of music episodes on level-2, although this only turned out to be a significant predictor in one model (Model 2a). A total of 70 measurements were available per participant (five time points per day for 14 consecutive days), making a total of 2100 possible observations. As HLM uses a listwise deletion procedure in the case of missing values, the degrees of freedom (df) vary between the tested models. In models concerning subjective reports of stress, a total of 1883 observations were entered into the analyses. With regard to neuroendocrine and autonomic markers of stress, a total of 1773 observations were entered into the analyses. As music listening occurred in 21.2% of daily assessments, the number of degrees of freedom was further reduced to 412 when subjective stress reports were considered as the outcome and 379 when biological markers of stress were considered as the outcome, respectively. As neither sCort nor sAA was normally distributed, we natural log-transformed both sCort and sAA [ln (*x*) + 10]. Two participants had not listened to music at all during the 14-day period of assessment, and were therefore not entered into analyses in which valence/arousal as well as reasons for music listening were examined.

Hypothesis testing was performed in accordance with Woltman et al. (2012). Therefore, both unconditional and conditional models were specified. The comparison between these models was undertaken by means of χ^2^- statistics, comparing the reduction in deviance as a measure of model fit. As an indicator of explained variance, pseudo-*R*^2^ is reported, calculated in accordance with the formula [(σ^2^(unconditional growth model) - σ^2^(subsequent model))/σ^2^(unconditional growth model)] (Singer and Willett, 2003). Mediation analyses were performed using the stepwise procedure as recommended by Kenny et al. (2003), Korchmaros and Kenny (unpublished).

*P*-values of ≤0.05 were considered significant. For all analyses, unstandardized coefficients (UC) are reported.

## Results

Participants reported having suffered from FMS for a mean of 120 ± 86 months. The mean BMI was 25.24 ± 2.90. Four participants had conditions which potentially influenced sCort or sAA: one had a BMI of 31.2, one suffered from Hashimoto’s thyroiditis, and two suffered from inflammatory respiratory diseases. As there is no reason to assume that these conditions affect subjective data, these participants were included in all analyses regarding subjective data. For analyses in which biological markers were included, we initially excluded these four participants, although as their exclusion did not alter the pattern of results, we decided to include these patients in all analyses for the sake of consistency.

Data from the MPQ (*n* = 29) on participants’ habitual music listening behavior revealed that patients listened to music on average for 2.0 ± 2.2 h per day. The reasons most commonly stated for listening to music were: ‘activation’ (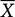 = 3.62), ‘relaxation’ (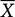 = 3.52), and ‘distraction’ (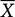 = 3.31). Concerning the importance of music for their lives, on a scale ranging from 1 (‘not at all important’) to 5 (‘very important’), patients reported a moderate level of importance (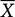 = 3.38 ± 1.37). A total of seven participants (=24.1%) reported that they were currently actively making music. Half of the participants (*n* = 15) stated that they had actively made music in the past.

With regard to the ecological momentary assessment data, music listening was reported in 21.2% of daily assessments. The music that was listened to was rated as rather positive in valance (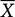 = 72.9 ± 14.1) and high in arousal (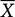 = 61.42 ± 22.7). The reasons most commonly stated for music listening were: ‘relaxation’ (48.8%), ‘distraction’ (34.5%), ‘activation’ (25.8%), and ‘reducing boredom’ (12.3%).

The overall mean pain intensity, rated on a scale ranging from 0 to 100, was 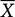 = 47.5 ± 25.0. On a scale from 0 to 5, patients rated a moderate level of perceived control over pain 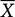 = 2.82 ± 1.1. Perceived stress was rated with a mean of 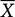 = 1.5 ± 1.0 on a scale from 0 to 4.

### Does Music Listening in Daily Life Reduce Perceived Pain Intensity (Hypothesis 1)?

We first examined whether music listening was associated with reduced levels of perceived pain intensity (Model 1a). The unconditional model included only perceived pain intensity as dependent variable at level-1 and number of music episodes at level-2, and no music listening. We then specified the following conditional model: perceived pain intensity levels were modeled as a function of music listening (yes/no; β_1j_) and a residual component (*r*_ij_). At the individual level (level-2), both the intercept (β_0_) and the slope (β_1_) were modeled as a function of number of music episodes (γ_01,_ γ_11_) and a residual component (u_0_). However, there were no associations between music listening and perceived pain intensity [*UC* = 1.64, *t*(1918) = 1.001, *p* = 0.317]. As music listening *per se* did not affect pain intensity, we tested whether music characteristics, i.e., perceived valence and arousal of the selected music, influenced perceived pain intensity (Model 1b). We specified the following conditional model: perceived pain intensity levels were modeled as a function of valence (0–100) (β_1j_), arousal (0–100) (β_2j_), and a residual component (*r*_ij_). At the individual level (level-2), the intercept (β_0_) was modeled as a function of number of music episodes (γ_01_) and a residual component (u_0_). Neither valence [*UC* = -0.03, *t*(384) = -0.328, *p* = 0.743] nor arousal [*UC* = -0.09, *t*(384) = -1.221, *p* = 0.223] was associated with any changes in perceived pain intensity. Concerning the reasons for music listening (Model 1c, **Table 1**), ‘activation’ was the only reason associated with a reduction in perceived pain intensity. The reduction in deviance (from the model including ‘relaxation,’ ‘distraction,’ and ‘reducing boredom’ as predictors to the model additionally including ‘activation’ as predictor) was marginally significant (χ^2^ = 12.11; df = 6; *p* = 0.059), with ‘activation’ explaining 1.86% of the variance in perceived pain intensity.

**Table 1:** Hierarchical linear models predicting repeatedly measured perceived pain intensity (70 measures; Model 1c), repeatedly measured perceived control over pain (70 measures; Model 2c) as well as repeatedly measured subjective stress (70 measures; Model 3c) by reasons for music listening using full maximum likelihood.

	Model 1c: changes in pain intensity by reason for music listening		Model 2c: changes in control over pain by reason for music listening		Model 3c: changes in subjective stress by reason for music listening	
Fixed effects	UC^1^	SE^2^ (df), *p*	UC^1^	SE (df), *p*	UC^1^	SE (df), *p*
Intercept level-2	53.76	6.68 (26), ≤*0*.*001*	2.82	0.29 (25), ≤*0*.*001*	1.49	0.28 (26), *≤0*.*001*
Number of music episodes^4^	-0.10	0.31 (26), *0.752*	0.01	0.01 (25), *0.645*	0.01	0.01 (26), *0.498*
Relaxation^5^ (0/1)	-1.07	2.66 (382), *0.688*	0.29	0.08 (290), ≤*0*.*001*	-0.29	0.17 (382), *0.085*
Activation^5^ (0/1)	-5.49	2.63 (382), *0.038*	0.29	0.08 (290), ≤*0*.*001*	-0.29	0.14 (382), *0.037*
Distraction^5^ (0/1)	-0.66	2.39 (382), *0.781*	-0.14	0.07 (290), *0*.*065*	0.15	0.12 (382), *0.209*
Reducing boredom^5^ (0/1)	-4.23	3.24 (382), *0.192*	0.16	0.24 (290), *0.503*	-0.10	0.30 (382), *0.735*

**Random effects**	**VC^**6**^**	**SD^**3**^ (df), *p***	**VC^**6**^**	**SD (df), *p***	**VC^**6**^**	**SD (df), *p***

Intercept level-1	467.48	21.64 (26), ≤*0.001*	0.73	0.85 (25), ≤*0.001*	0.37	0.61 (26), *≤0*.*001*
Residual	203.87	14.28	0.37	0.61	0.63	0.80

*^1^UC, Unstandardized Coefficients. ^2^SE, Standard Error. ^3^SD, Standard Deviation. ^4^Number of music episodes: total number of music episodes per participant per measurement period (0–53). ^5^(0/1): 0 = no, 1 = yes. ^6^VC, Variance Component*.

### Does Music Listening in Daily Life Increase Control Over Pain (Hypothesis 2)?

Next, we examined whether music listening was associated with increased levels of perceived control over pain. The unconditional model included only perceived control over pain as dependent variable at level-1 and number of music episodes at level-2, and no music listening. The conditional model (Model 2a) examined whether the level of perceived control over pain varied as a function of music listening (yes/no; β_1j_) and a residual component (*r*_ij_) at level-1. At the individual level (level-2), both the intercept (β_0_) and the slope (β_1_) were modeled as function of number of music episodes (γ_01_, γ_11_), and a residual component (u_0_). In line with our expectations, music listening was associated with higher levels of perceived control over pain [*UC* = 0.30, *t*(1458) = 3.548, *p* < 0.001]. Furthermore, the number of music episodes influenced the association between perceived control over pain and music listening [*UC* = 0.01, *t*(1458) = 2.047, *p* = 0.041], with those reporting more music episodes experiencing more control over pain. The reduction in deviance was significant (χ^2^ = 14.59; df = 4; *p* = 0.006), with 0.83% of the variance in control over pain being explained by our predictors.

We then examined whether perceived control over pain varied as a function of the perceived valence and arousal of the selected music (Model 2b). Therefore, we specified a conditional model with valence (0–100) and arousal (0–100) being included as predictors at level-1, and at level-2 the intercept was modeled as a function of number of music episodes. Valence [*UC* = 0.01, *t*(292) = 2.719, *p* = 0.007], but not arousal [*UC* = 0.00, *t*(292) = 0.285, *p* = 0.776], was associated with higher levels of perceived control over pain. The reduction in deviance was significant (χ^2^ = 10.78, df = 4, *p* = 0.029), with 2.35% of the variance in perceived control over pain being explained. Next, we examined whether reasons for music listening were associated with the level of perceived control over pain. Therefore, we specified a conditional model in which the reasons for music listening [‘relaxation’ (yes/no), ‘activation’ (yes/no), ‘distraction’ (yes/no), and ‘reducing boredom’ (yes/no)] were included as predictors at level-1 (Model 2c, **Table 1**). We found that ‘relaxation’ and ‘activation’ were associated with an increase in perceived control over pain, with 3.79% of the variance in control over pain being explained by reasons for music listening (χ^2^ = 17.08, df = 6, *p* = 0.009).

### Does Music Listening in Daily Life Reduce Stress in Patients with FMS (Hypothesis 3)?

#### Subjective Stress

We examined whether music listening was associated with reduced levels of subjective stress (Model 3a). The unconditional model included only subjective stress as dependent variable at level-1 and number of music episodes and a residual component at level-2, and no music listening. Concerning the conditional model (Model 3a) subjective stress levels were modeled as a function of music listening (yes/no; β_1j_) and a residual component (*r*_ij_). At the individual level (level-2), both the intercept (β_0_) and the slope (β_1_) were modeled as a function of number of music episodes (γ_01_, γ_11_) and a residual component (u_0_). There was no association between music listening and subjective stress [*UC* = -0.12, *t*(1918) = -1.204, *p* = 0.229]. Subsequently, we examined whether valence or arousal were associated with subjective stress, adjusting the conditional model accordingly with valence and arousal at level-1, and only the intercept being modeled as a function of number of music episodes at level-2 (Model 3b). However, neither valence [*UC* = -0.00, *t*(384) = -0.855, *p* = 0.393] nor arousal [*UC* = 0.00, *t*(384) = 0.752, *p* = 0.453] was associated with subjective stress. Concerning the reasons for music listening (Model 3c, **Table 1**), ‘activation’ was the only reason for music listening which was associated with lower subjective stress levels. The reduction in deviance (calculated as the reduction in deviance from the unconditional model to the conditional model) was significant (χ^2^ = 12.75, *df* = 6, *p* = 0.047), with 2.42% of the variance being explained by reasons for music listening.

#### Physiological Parameters of Stress

Music listening was not associated either with sCort [*UC* = -0.06, *t*(1816) = -0.694, *p* = 0.488] or with sAA [*UC* = -0.01, *t*(1807) = -0.078, *p* = 0.938]. Furthermore, valence and arousal ratings did not affect sCort concentrations [valence: *UC* = 0.00, *t*(352) = 1.034, *p* = 0.302; arousal: *UC* = 0.00, *t*(352) = -1.244, *p* = 0.214] or sAA activity [valence: *UC* = 0.00, *t*(352) = 0.927, *p* = 0.354; arousal: *UC* = 0.00, *t*(352) = 0.743, *p* = 0.458]. None of the reasons for music listening was associated with sCort secretion [*UC* < 0.11, *t*(350) < 1.092, *p* > 0.276], as was the case with sAA activity [*UC* < -0.11, *t*(350) < -0.789, *p* > 0.431].

### Is the Pain-Reducing Effect of Music Listening Mediated by the Biological Stress-Responsive Systems (Hypothesis 4)?

As our analyses above identified relations among control over pain, the reason of ‘relaxation,’ and the reason of ‘activation,’ we only tested whether the increase in perceived control over pain when music was listened to for the reason of ‘relaxation’ and ‘activation’ was mediated by the biological stress-responsive systems. However, as none of the reasons for music listening was associated either with sAA or with sCort [see Does Music Listening in Daily Life Reduce Stress in Patients with FMS (Hypothesis 3)?], conditions for mediation analyses were not met and we therefore refrained from performing mediation analyses.

## Discussion

We found a beneficial effect of listening to music on how FMS patients coped with pain in daily life: whereas the perceived pain intensity was not affected by listening to music, perceived control over pain was significantly increased after having listened to music. This effect was especially profound in participants who listened to music more often, with an increase in the number of music episodes being associated with an increase in the pain-reducing effect of music listening. The perceived valence and the reasons for music listening (‘relaxation’ and ‘activation’ emerged as the most important factors) seem to be especially relevant, as they predicted increases in control over pain. We did not find a specific stress-reducing effect of music in FMS patients. The reason of ‘activation’ again predicted successful reduction in subjective stress, but the biological stress-responsive systems were not affected by music listening in daily life in these patients. Thus, the pain-reducing effect of music listening did not prove to be mediated by a reduction in levels of stress biomarkers.

Mirroring the heterogeneity in findings on the effects of music listening on pain intensity (Mitchell and MacDonald, 2012), we were not able to find an effect of music listening on pain intensity in daily life. It is assumed that music listening exerts its effects in the central nervous system, which then triggers emotional and cognitive processes as well as alterations in the peripheral nervous system (Koelsch, 2014). Evidence supports the notion that music-induced analgesia is produced by the central nervous system, but does not translate into the peripheral nervous system, i.e., by affecting nociceptive receptors (Garza-Villarreal et al., 2014). Especially as FMS is characterized by a sensitization to pain combined with dysregulated pain-inhibitory pathways, our results support the notion that mere music listening in daily life does not yield improvements in pain intensity in patients with FMS. Although Onieva-Zafra et al. (2013) did find an effect of music listening on pain intensity in patients with FMS, these patients were instructed to listen to music at least once a day for 30 min in the context of a trial. In our study, participants did not receive any instructions regarding their music listening. Our sample listened to music once a day on average while going on with their daily lives; half of our participants listened to music more than once a day and the other half listened to music less than once a day. The effects of an intervention in which patients are guided to listen to music on a daily basis might therefore be beyond the effects of our study, as we did not manipulate the music listening behavior of our participants, but rather studied the short-term effects of music listening as it happens in daily life. More profound effects of music listening might be observable if people were specifically instructed to engage with music on a regular basis.

Pothoulaki et al. (2012) argue that music interventions cannot affect physiological processes in chronic diseases but can yield improvements in quality of life, with increasing perceived control as one mechanism through which music listening has health-beneficial outcomes. Turner et al. (2007) support this notion, as they showed that improvements in pain coping as achieved by cognitive-behavioral therapy (CBT) are mostly mediated by increases in control over pain. We were able to show that control over pain was improved after listening to music – especially for those who reported listening to music more regularly. It is assumed that characteristics both of the music and of the listener contribute to the pain-reducing effect of music listening (Mitchell and MacDonald, 2012; Pothoulaki et al., 2012). Here, we replicated the finding that music rated as high in valence was effective in reducing pain (Roy et al., 2008). However, the arousal of the music did not play a major role, in contrast to evidence from previous studies in which music low in arousal was used as a stimulus (Garza-Villarreal et al., 2014; Picard et al., 2014). To the best of our knowledge, we were the first to show that reasons for music listening seem to be especially relevant to the health-beneficial effect of music listening (Linnemann et al., 2015a). In the current study, especially ‘activation’ and ‘relaxation’ predicted an increase in control over pain, suggesting that music listening might lead to successful pain management. At first glance, these two reasons for music listening seem to be contradictory, but these findings can be reconciled: In the management of pain, both relaxation and activation are well-known strategies that have the potential to reduce pain. With regard to activation, CBT therapists instruct patients to reduce avoidance behavior as this is thought to maintain the persistence of chronic pain (Philips, 1987; McCracken and Samuel, 2007). On the other hand, relaxation techniques were shown to be associated with improved coping with pain (Turner et al., 2007). Therefore, both activation and relaxation can lead to improvements in pain management. Interestingly enough, the reason of ‘distraction’ was not found to decrease pain in our study. This is striking, especially as distraction is thought to be a major contributor to music-induced analgesia (Mitchell and MacDonald, 2012; Pothoulaki et al., 2012). Although we cannot rule out that patients actually perceived distraction while pursuing activation and relaxation when listening to music, distraction in daily life seems to be inefficient for coping with pain in chronic pain patients.

Our model proposes that the health-beneficial effect of listening to music is mediated by a reduction in stress (Thoma and Nater, 2011). There is evidence in the literature that the stress- and pain-reducing effects of listening to music are inter-related (Garza-Villarreal et al., 2014). Some experimental studies have shown that music listening affects both pain and stress (Good and Ahn, 2008; Bauer et al., 2011). However, others have demonstrated that music listening exclusively affects pain management and does not affect stress (Good et al., 2013). Nevertheless, these studies were predominantly conducted in experimental settings, examining acute pain either in healthy controls or in patients undergoing surgery. Furthermore, patients had to listen to music that was mostly chosen by the experimenter. This procedure is questionable, as it is known from the literature that self-selected music exerts the greatest stress-reducing effects (Chanda and Levitin, 2013). Therefore, we chose not to influence the music listening behavior of our participants but examined the effects of mere music listening in daily life. In our study, the pain-reducing effect of listening to music was not mediated by biological stress-responsive systems. This might be due to the already distorted ANS and HPA axis functioning in patients with FMS (Martinez-Lavin, 2002; Tak et al., 2011). Our results suggest that mere music listening may not be capable of impacting these chronic alterations in HPA and ANS functioning. Nevertheless, it remains open to further investigation whether, by means of event-based sampling methods, effects of music listening on acute stressors might be found. Furthermore, it might be possible that a more intense engagement with music (i.e., more music listening, active music-making) might be necessary in order to affect these systems in the body. In this context, music intervention studies set in daily life should examine whether the disease-induced alterations in HPA and ANS functioning can be positively affected.

Although this is the first study to examine the effects of music listening on pain and stress in patients with FMS in an everyday life setting, combining both psychological and biological outcomes, this study is not without limitations. First, the timing of the assessment relative to the music listening does not allow us to draw any conclusions about immediate effects of music listening on pain and stress. As there were, in part, up to 4 h between music listening and the assessments, we cannot rule out that music listening had acute effects on subjective pain and stress reports which were not sufficiently persistent to be captured by the later assessment. Second, we only asked about deliberate music listening. Thus, we cannot rule out that participants were exposed to background music during episodes that we coded as no-music episodes. Future studies are necessary to examine the effects of background music on health-related outcomes. Third, our sample size was moderate, although we did use a repeated measures design, which leads to a high number of observations per participant. Fourth, our hypotheses are based on within-person observations. A comparison group (i.e., participants with acute pain and/or healthy participants) would allow the testing of between-subject hypotheses that examine the potentially different mechanisms underlying the pain-reducing effects of music listening between a population with chronic pain versus a population with acute pain. Finally, as we only assessed women, our results do not allow generalizations to male FMS patients. This should be a focus of future research.

## Conclusion

Fibromyalgia syndrome is a complex and in part poorly understood chronic pain disorder. An optimal management of pain is not yet available, although a multimodal approach including music interventions has been suggested (Onieva-Zafra et al., 2013; Picard et al., 2014). We were able to show that mere music listening in daily life has beneficial effects on control over pain. It seems to be relevant why one listens to music, as in our study, listening to music for the reason of ‘activation’ or ‘relaxation’ predicted successful pain coping. We did not find this pain-reducing effect to be mediated by stress-responsive systems. Future studies need to examine acute effects of music listening on pain and stress by means of event-based sampling methods. Furthermore, it remains open to further investigation whether a more intense engagement with music in daily life can positively impact these stress-responsive systems by means of music interventions in daily life.

## Author Contributions

All authors contributed to the study design. Data collection was performed by MK, SF, and JD. JS and SF conducted the bio-chemical analyses of saliva samples AL, MK, JS, and UN performed the data analysis and interpretation. AL and UN drafted the manuscript, and MK, JS, SF, and JD provided critical revisions. All authors approved the final version of the manuscript for submission. All authors agree to be accountable for all aspects of the work ensuring that questions related to the accuracy or integrity of any part of the work are appropriately investigated and resolved.

## Conflict of Interest Statement

The authors declare that the research was conducted in the absence of any commercial or financial relationships that could be construed as a potential conflict of interest.
